# Food expenditure, income, and mental health: Outcomes from the UK Household Longitudinal Survey

**DOI:** 10.1371/journal.pone.0308987

**Published:** 2024-09-04

**Authors:** Muhammad Waqas, Syka Iqbal, Barbara J. Stewart-Knox

**Affiliations:** 1 Department of Accounting, Finance and Economics, University of Bradford, Bradford, United Kingdom; 2 Department of Psychology, University of Bradford, Bradford, United Kingdom; Imperial College London, UNITED KINGDOM OF GREAT BRITAIN AND NORTHERN IRELAND

## Abstract

The incidence of mental health problems is increasing in the United Kingdom and may be associated with lower dietary quality. Food expenditure is a marker of food insecurity with potential implications for mental health. This analysis considers data collected as part of the United Kingdom Household Longitudinal Survey (UKHLS), also known as ‘Understanding Society’ (2009–2021) (N = 388,944) to determine the extent to which food expenditure within and outside the household, is associated with mental health, whilst controlling for demographic factors. Mental health was measured using the General Health Questionnaire (GHQ-12) for which responses were on a 4-point scale and reverse-scored so that a higher score represented more favourable mental health. Household food expenditure and food expenditure outside the home were the outcomes. Controlling for socioeconomic and demographic factors, fixed-effects models indicated that better mental health was associated with greater household food expenditure and with greater food expenditure outside the home and that this association persisted post-lockdown. Among those on lower incomes better mental health was associated with lower food expenditure. When people who identified as white and non-white were modelled separately, better mental health was associated with lower food expenditure within and beyond the household only in those who identified as white. These findings imply that the mental health of people residing in the UK, particularly those on lower incomes and those who identify as white, may benefit from spending less of the household budget on food. In achieving United Nations General Assembly (2012) Sustainable Development Goals related to poverty, hunger and in promoting mental health, policies are needed to render food more affordable and to reduce other aspects of expenditure that impact upon food budgeting.

## 1. Introduction

The incidence of mental health problems is increasing in the United Kingdom (UK). In 2020, approximately 19% of adults reported experiencing symptoms of depression, a figure that rose to 21% by early 2021 [1, 2]. In the UK, mental health costs the NHS £15 million annually, with an estimated £100 billion to wider society owing to impact upon individuals’ ability to work [3]. This upward trend highlights the importance of understanding the factors associated with mental health so that potential targets for intervention and policy may be developed. This secondary analysis of UK Household Longitudinal Survey (UKHLS) and Understanding Society (2009–2021) data, therefore, has taken mental health (GHQ score) of the head of household as the key variable. The UKHLS and Understanding Society surveys assessed mental health using the General Health Questionnaire (GHQ-12) [4, 5]. The GHQ-12 is a short, self-reported overall measure of mental health, which has been shown to have good internal consistency [6], discriminate ability [7] and has been deemed suitable for assessment of population mental health [8].

Mental health measured using the GHQ-12 tends to be less favourable among those on lower incomes [9–12] and varies between demographic groups. Analysis of the British Household Panel Survey (BHPS) (1991–2008) and Understanding Society data up to 2012 and more recently in 2020 [12] suggested that poorer mental health (GHQ) was associated with being female, younger, single, having fewer children, lower education attainment and precarious employment [13]. This indicates the importance of controlling for socio-economic factors when seeking to understand mental health. An affordable, nutritious diet is considered a prerequisite for good mental health, yet diets meeting UK healthy eating recommendations such as the EATWELL guidelines [14], can be up to 17% more expensive [15]. The Covid-19 pandemic and associated lockdown brought about increases in the cost of food which along with rising energy bills, housing and transport costs, have put further pressure upon household budgets that may have impacted upon food purchase [16, 17]. UK household food expenditure, the amount spent by a household on food items, increased by 3.9% between 2009–2020 when it constituted approximately 10% of the household budget [16]. Despite a 9.9% decline in household expenditure during 2020 as a consequence of reduced activity outside the home during lockdown, household expenditure rose sharply during 2021 to 3.6% above pre-pandemic levels [1, 18]. Household food insecurity, which according to Department for Environment, Food and Foreign Affairs (DEFRA) [16], is the inability to access safe, healthy, affordable food [16, 19], also increased during the pandemic [19]. The UK Food Standards Agency consumer tracker survey of 2000 respondents aged 16–75 years living in England, Wales and Northern Ireland during 2023 has implied increasing food insecurity with 86% of respondents expressing concern about rising food prices and 25% unable to meet the cost of essential food items [20]. Food expenditure surveys are considered a good indicator of food security [21] and food expenditure tends to be lower in food insecure households [22]. Given the putative link between food insecurity and food expenditure [21, 22], and that problems meeting household expenditure can be associated with poorer mental health [13, 23, 24], food expenditure has been taken as the variable of interest in this analysis. For the purpose of this analysis, food expenditure has been defined as the amount of money spent by the houshold on food purchased in shops and the amount spent on food purchesed outside the home in catering outlets [25].

The impact of higher food expenditure upon mental health is likely to be greater among those residing in households with lower disposable income [10, 26]. This analysis will therefore test for interaction between food expenditure and income in explaining mental health. Given evidence for differences between white and non-white ethnicities in mental health [27], eating practices [28] and food expenditure [29] additional separate analyses have been conducted on people identifying as white and non-white [30]. Previous analysis of GHQ scores derived from the UK Household Longitudinal Survey (HLS) indicated that mental health declined between 2019 and the onset of the pandemic [31] and with successive lockdowns [32–34]. This analysis, therefore, has compared responses before and during lockdown.

Understanding how mental health relates to food expenditure is important given it has implications for the achievement of several of the United Nations (UN) 2030 Sustainable Development Goals (SDGs): 1. No Poverty; 2. Zero Hunger; 3. Good Health; 10. Reduce Inequalities [35]. This analysis will investigate the extent to which food expenditure, both within and outside the household, is associated with mental health, pre and post pandemic, taking income into account and comparing people with white and non-white identity. It is predicted that food expenditure will be associated with mental health and that this association will be stronger among those with a lower income. It is also predicted that there will be differences in the association between mental health and expenditure before and during lockdown and in those of white and non-white identity.

## 2. Method

### 2.1 Sampling

This analysis considers data collected as part of the United Kingdom Household Longitudinal Survey (UKHLS), also known as ‘Understanding Society’, which collects data from a UK nationally representative sample on an annual basis. These data are publicly available to anyone and can be downloaded after registering at the UK Data Service website (https://ukdataservice.ac.uk/). These data are stored under SN6614 at the UK Data Service website. The UKHLS contains information from approximately 50,000 individuals in each wave. Data for ‘mainstage waves’ 1–11 collected from 2009 to 2021 (N = 389,150) were used in this analysis (please see Tables 1 and 2 below).

**Table 1:** General Health Questionnaire (GHQ) scores and demographic factors (N = 389,150).

	Percentage	Number of observations	Std. dev. GHQ	Mean GHQ Score
Male	44.22	172,088	5.17	25.47
Female	55.78	217,062	5.75	24.36
Pre-lockdown	97.53	379,534	5.52	24.87
Post-lockdown	2.47	9,616	5.70	24.00
Higher degree	38.06	148,144	5.23	25.14
Other degree	49.36	192,072	5.64	24.75
No qualification	11.43	44,461	5.90	24.30
Single	19.83	77,148	6.06	24.44
Married	65.75	255,869	5.19	25.15
Divorced	8.49	33,020	6.47	23.52
Widowed	5.93	23,078	5.40	24.87
No children	72.19	280,919	5.53	24.92
One child	11.91	46,382	5.64	24.55
Two children	11.43	44,475	5.32	24.87
Three+ children	4.47	17,374	5.72	24.54
Self-employed	7.85	30,528	4.78	25.65
Employed	49.36	192,100	5.06	25.22
Unemployed	4.58	17,821	7.06	22.42
Inactive	36.89	143,537	5.92	24.50
Other (retired; student etc)	1.33	5,164	5.83	24.52
Northern England	37.68	146,620	5.65	24.69
Southern England	40.51	157,625	5.42	24.98
Wales	6.83	26,567	5.63	24.58
Scotland	8.83	34,373	5.48	25.06
Northern Ireland	6.16	23,965	5.43	25.01
Wave 19 2009–11	9.76	37,992	5.36	24.90
Wave 20 2010–12	10.70	41,642	5.53	24.75
Wave 21 2011–13	10.04	39,080	5.51	24.91
Wave 22 2012–14	9.58	37,279	5.58	24.98
Wave 23 2013–15	9.17	35,688	5.63	24.81
Wave 24 2014–16	9.59	37,309	5.44	25.13
Wave 25 2015–17	9.20	35,812	5.47	25.06
Wave 26 2016–18	8.80	34,230	5.53	24.89
Wave 27 2017–19	8.06	31,370	5.58	24.75
Wave 28 2018–20	7.75	30,152	5.55	24.66
Wave 29 2019–21	7.35	28,596	5.63	24.37
Income 1st-25th percentile	25.00	97,288	6.11	24.15
Income 26th-50th percentile	25.00	97,287	5.69	24.65
Income 51st-75th percentile	25.00	97,288	5.26	25.15
Income 76th percentile +	25.00	97,287	4.89	25.45
White identity	85.23	329,057	5.45	24.87
Non-White identity	14.77	57,010	5.95	24.73
Total sample	100.00	389,150	5.53	24.85

**Table 2:** General Health Questionnaire (GHQ) scores, income and age (N = 389,150).

	Mean	Std. dev.	Min	Max
Income (logged)	7.99	0.76	0	14.83
Age	49.07	17.74	18	120
GHQ Score	24.85	5.53	0	36

### 2.2 Procedure

Interviews were typically conducted face-to-face in respondents’ homes facilitated by trained interviewers or by the respondents themselves completing their survey online. Every section of the questionnaire, including all the questions, were answered voluntarily. Further information about the UKHLS survey such as, study design, sampling, study timeline, questionnaire design, interview process, fieldwork procedures, response rates, data collection and data processing can be accessed at the following address: https://www.understandingsociety.ac.uk/documentation/mainstage/user-guides/main-survey-user-guide/.

### 2.3 Measures

The key outcome variable was mental health measured using the 12-item General Health Questionnaire (GHQ-12) [4, 5], which was completed during each wave of data collection considered. The GHQ is based on responses to 12 separate questions each of which was scored on a four-point scale. Scores were then summed producing an overall GHQ score that ranged from 0 to 36. For ease of interpretation, we reversed the overall score so that a value of 36 represented the highest level and henceforth we have referred to this variable as mental health or mental well-being.

The variable of interest was food expenditure derived from two questions included in all waves derived for analysis: "About how much has your household spent in total on food and groceries in the last four weeks from a supermarket or other food shop or market?… ."; "About how much have you and other members of your household spent in total on meals, snacks or non-alcoholic drinks purchased outside the home in the last four weeks? …."

### 2.4 Data analysis

This analysis has examined the relationship between food expenditure and mental health using the GHQ-12. The analysis was repeated having sub-divided white and non-white respondents. Separate models were created each for household food expenditure and food expenditure outside the home: 1) uncontrolled model of GHQ scores; 2) model controlled for individual fixed effects (demographic factors) in the total sample (N = 388,944); 3) model controlled for demographic factors in respondents reporting a white identity (n = 330,052); model controlled for demographic factors in of those identifying as non-white (n = 55,179).

All analyses were conducted using STATA 17. Fixed effects panel models were constructed to explain the key outcome variable of mental health, taking income into account, and associations with the explanatory variables of household food expenditure and food expenditure outside the home. Fixed Effects models were run with different specifications. Our analysis assumes that mental health (MH_it_) surveyed at time t is associated with his/her food expenditure represented by (X_it_). To ensure that aggregate time series variation is completely absorbed, we add wave dummies w_t_ to our specification. We also included region dummies (r) and a vector of time variant individual level controls (C_it_) (Tables 3 and 4). We used robust standard errors clustered at the individual level. This yields the following explanatory model where a, w, and r are the individual, wave, and region fixed effects respectively and ɛ_it_ is the error term:

$${MH}_{it}=\beta_{0}+\beta_{1}X_{it}+\beta_{2}C_{it}{+a}_{i}+w_{t}+r+\varepsilon_{it}$$

**Table 3:** General Health Questionnaire (GHQ) scores, demographic factors, and coefficient for household food expenditure (fixed effects panel model including income and lockdown interactions).

Demographic Variables	Uncontrolled	Controlled	White Identity	Non-White Identity
Food Expenditure (log)	-0.13***	0.95***	0.96***	0.78
	(0.04)	(0.19)	(0.21)	(0.44)
FoodExpend(log)#Income(log)	0.03***	-0.11***	-0.11***	-0.09
	(0.00)	(0.02)	(0.03)	(0.06)
Lockdown#FoodExpend(log)	-0.17***	-0.18**	-0.23**	-0.03
	(0.01)	(0.09)	(0.10)	(0.20)
Income (log)		0.71***	0.70***	0.69**
		(0.13)	(0.15)	(0.31)
Lockdown		0.50	0.69	-0.03
(ref = post-23/3/20)		(0.52)	(0.58)	(1.13)
Sex (Male)		-0.79	-0.66	-0.24
(ref = female)		(0.61)	(0.66)	(1.18)
Age		0.06	0.04	0.14
		(0.04)	(0.04)	(0.09)
University degree		-0.90***	-0.73***	-1.12***
(ref = no qualification)		(0.14)	(0.17)	(0.32)
Other qualifications		-0.40***	-0.27	-0.34
		(0.12)	(0.14)	(0.26)
Married		-0.04	-0.03	-0.05
(ref = single)		(0.06)	(0.07)	(0.17)
Divorced		-0.49***	-0.49***	-0.49**
		(0.09)	(0.10)	(0.22)
Widowed		-0.54***	-0.50***	-0.97***
		(0.12)	(0.12)	(0.37)
One child		-0.13***	-0.13***	-0.21
(ref = zero child)		(0.04)	(0.05)	(0.12)
Two children		-0.23***	-0.24***	-0.20
		(0.06)	(0.06)	(0.15)
Three children		-0.20**	-0.20	-0.20
		(0.09)	(0.10)	(0.20)
Self-employed		0.14***	0.20***	-0.15
(ref = employed)		(0.05)	(0.06)	(0.14)
Unemployed		-1.36***	-1.34***	-1.49***
		(0.06)	(0.07)	(0.14)
Inactive (student/retired/etc)		-0.34***	-0.30***	-0.53***
		(0.04)	(0.04)	(0.11)
Other employed (unpaid)		0.01	0.06	-0.37
		(0.08)	(0.08)	(0.21)
Northern England		0.16	0.18	0.11
(ref = Southern England)		(0.14)	(0.15)	(0.41)
Wales		0.33	0.25	2.50
		(0.29)	(0.30)	(1.87)
Scotland		0.01	-0.17	0.08
		(0.33)	(0.34)	(1.04)
Northern Ireland		1.19**	1.29	-0.44
		(0.60)	(0.71)	(2.28)
20.wave 2010–12		-0.21***	-0.20***	-0.28**
(ref = wave 19 2009–11)		(0.05)	(0.05)	(0.13)
21.wave 2011–13		-0.17	-0.12	-0.47**
		(0.09)	(0.09)	(0.21)
22.wave 2012–14		-0.17	-0.13	-0.44
		(0.13)	(0.13)	(0.30)
23.wave 2013–15		-0.41**	-0.37**	-0.76
		(0.17)	(0.17)	(0.40)
24.wave 2014–16		-0.19	-0.11	-0.74
		(0.21)	(0.21)	(0.49)
25.wave 2015–17		-0.31	-0.23	-0.83
		(0.25)	(0.26)	(0.58)
26.wave 2016–18		-0.59**	-0.52	-1.09
		(0.29)	(0.30)	(0.67)
27.wave 2017–19		-0.78**	-0.68**	-1.40
		(0.33)	(0.34)	(0.77)
28.wave 2018–20		-0.92**	-0.80**	-1.71**
		(0.37)	(0.38)	(0.86)
29.wave 2019–21		-1.07***	-0.94**	-2.00**
		(0.41)	(0.42)	(0.96)
Constant	24.22***	17.44***	17.73***	15.55***
	(0.12)	(2.08)	(2.24)	(4.23)
Observations	389,770	388,944	330,052	55,179
R-squared	0.00	0.01	0.01	0.01
Number of Panels	75,919	75,867	60,368	14,681

Robust standard errors are presented within parentheses, underneath the beta coefficients. Levels of significance

*** p<0.01

**p<0.05

The uncontrolled model in column 2 only has logged food expenditure and its interactions with income and lockdown as X variables. In contrast, controlled models include additional controls: logged income, lockdown dummy, gender, age, qualification dummies, marital status dummies, number of children dummies, labour market status, geographical region and wave dummies.

Food expenditure and income interaction is presented in row 3 and food expenditure and lockdown interaction is presented in row 4.

**Table 4:** General Health Questionnaire (GHQ) scores, demographic factors, and coefficient for expenditure outside the home (fixed effects panel model including income and lockdown interactions).

Demographic Variables	Uncontrolled	Controlled	White Identity	Non-White Identity
Food Expenditure (log)	-0.13***	0.49***	0.51***	0.38
	(0.04)	(0.12)	(0.13)	(0.35)
FoodExpend(log)#Income(log)	0.02***	-0.05***	-0.05***	-0.05
	(0.00)	(0.02)	(0.02)	(0.04)
Lockdown#FoodExpend(log)	-0.23***	-0.06	-0.13	0.13
	(0.01)	(0.07)	(0.07)	(0.15)
Income (log)		0.29***	0.28***	0.36
		(0.07)	(0.07)	(0.19)
Lockdown		-0.30	-0.12	-0.71
(ref = post-23/3/20)		(0.28)	(0.31)	(0.66)
Sex (Male)		-0.37	0.16	-0.68
(ref = female)		(0.70)	(0.70)	(1.43)
Age		0.07	0.05	0.17
		(0.05)	(0.05)	(0.11)
University degree		-0.84***	-0.62***	-1.05***
(ref = no qualification)		(0.15)	(0.18)	(0.34)
Other qualifications		-0.42***	-0.25	-0.35
		(0.13)	(0.16)	(0.28)
Married		-0.03	-0.04	-0.05
(ref = single)		(0.07)	(0.07)	(0.18)
Divorced		-0.48***	-0.52***	-0.28
		(0.10)	(0.11)	(0.24)
Widowed		-0.49***	-0.47***	-0.90
		(0.13)	(0.14)	(0.47)
One child		-0.15***	-0.14***	-0.23
(ref = zero child)		(0.05)	(0.05)	(0.13)
Two children		-0.23***	-0.25***	-0.20
		(0.06)	(0.06)	(0.16)
Three children		-0.23**	-0.25**	-0.17
		(0.09)	(0.10)	(0.21)
Self-employed		0.14***	0.19***	-0.14
(ref = employed)		(0.05)	(0.06)	(0.15)
Unemployed		-1.32***	-1.33***	-1.38***
		(0.07)	(0.08)	(0.15)
Inactive (student/retired/etc)		-0.28***	-0.24***	-0.50***
		(0.04)	(0.05)	(0.11)
Other employed (unpaid)		0.04	0.08	-0.27
		(0.08)	(0.09)	(0.22)
Northern England		0.16	0.18	0.08
(ref = Southern England)		(0.15)	(0.16)	(0.44)
Wales		0.03	-0.02	1.82
		(0.30)	(0.31)	(2.13)
Scotland		-0.10	-0.31	0.04
		(0.35)	(0.36)	(1.17)
Northern Ireland		1.43**	1.62**	-1.07
		(0.60)	(0.69)	(2.35)
20.wave 2010–12		-0.16***	-0.15***	-0.23
(ref = wave 19 2009–11)		(0.06)	(0.06)	(0.15)
21.wave 2011–13		-0.16	-0.11	-0.51**
		(0.10)	(0.10)	(0.25)
22.wave 2012–14		-0.16	-0.12	-0.55
		(0.14)	(0.15)	(0.35)
23.wave 2013–15		-0.41**	-0.36	-0.91
		(0.19)	(0.19)	(0.46)
24.wave 2014–16		-0.21	-0.12	-0.92
		(0.24)	(0.24)	(0.57)
25.wave 2015–17		-0.37	-0.29	-1.01
		(0.28)	(0.29)	(0.68)
26.wave 2016–18		-0.63	-0.55	-1.34
		(0.33)	(0.33)	(0.79)
27.wave 2017–19		-0.86**	-0.75**	-1.74
		(0.37)	(0.38)	(0.90)
28.wave 2018–20		-1.03**	-0.90**	-2.11**
		(0.42)	(0.42)	(1.01)
29.wave 2019–21		-1.17**	-1.04**	-2.33**
		(0.47)	(0.47)	(1.12)
Constant	24.69***	20.52***	20.59***	17.65***
	(0.05)	(2.05)	(2.15)	(4.12)
Observations	340,019	339,317	287,828	48,087
R-squared	0.00	0.01	0.01	0.01
Number of pidp	71,657	71,606	57,046	13,765

Robust standard errors are presented within parentheses, underneath the beta coefficients. Levels of significance

*** p<0.01

**p<0.05

The uncontrolled model in column 2 only has logged food expenditure outside the home and its interactions with income and lockdown as X variable. In contrast, controlled models include additional controls: logged income, lockdown dummy, gender, age, qualification dummies, marital status dummies, number of children dummies, labour market status, geographical region and wave dummies.

Food expenditure and income interaction is presented in row 3 and food expenditure and lockdown interaction is presented in row 4.

The vector of time variant individual level controls included the log of household income, gender, age, education, marital status, number of children, and labour market status. We also included a pre/post-lockdown dummy to control for the lockdown effect. Gender was included as a dummy which takes value of 1 if male and 0 if female which rendered female the reference category. Age was included as a continuous variable. Three dummies were created for the education category, namely, “higher education”, “other education”, “no qualification”, where “higher education” includes university degree or any higher qualification, “other education” includes A-level, GCSE, or any other qualification less than university and “no qualification” dummy included respondents with no qualification and served as the reference category. Four dummies (single, married, divorced, widowed) were generated for the marital status variable with single as the reference category. For number of children, four categories were created: 0 child; 1 child; 2 children; 3 or more children with 0 child the reference category. For labour market status, four categories were created (self-employed, paid-employed, inactive, retired), where paid-employment was the reference category.

Our variable of interest was “food expenditure” represented by X_it_ in the above equation. Separate models were run on “household food expenditure” and “food expenditure outside home”. To test the relationship between food expenditure and mental health, we estimated the above equation initially by regressing MH_it_ on X_it_ alone to see base line estimates and then included a vector of controls.

We interacted the lockdown dummy with “food expenditure” variables (within and outside the household) in separate models to determine any change in the relationship between food expenditure and mental health pre- and post-lockdown. A lockdown dummy was created, which took the value of 1 if the interview had been conducted on or after 23 March 2020 (lockdown implementation date) and 0 if the interview took place before this date. This dummy was then multiplied with food expenditure variables in their respective models to obtain an interaction effect. Similarly, we interacted the “food expenditure” variables with “household income” to determine if change in relationships between “food expenditure” and mental health were associated with “household income”.

## 3. Results

### 3.1 GHQ and household food expenditure and food expenditure outside the home in the total sample (uncontrolled model)

The uncontrolled model explaining the key variable of mental health assessed using the General Health Questionnaire (GHQ) (5) in the total sample (N = 389,770) indicated that higher (more favourable) mental health (GHQ) scores were significantly (negatively) associated with lower household food expenditure (β = -0.13) (Table 3) and lower food expenditure outside the home (β = -0.13) (Table 4).

A significant interaction effect was observed between mental health (GHQ scores), income* and household food expenditure (β = 0.03) and expenditure outside the home (β = 0.03). Increase in income*food expenditure had a significant positive effect on mental health.

There was a significant interaction pre- and post-lockdown, such that there was a positive relationship between mental health (GHQ scores) and household food expenditure which changed to a negative relationship post-lockdown (β = -0.17) (Table 3). A significant interaction was also observed pre-lockdown for food expenditure outside the home with a positive association with mental health prior to lockdown which became non-significant post-lockdown (β = -0.23) (Table 4).

### 3.2 GHQ and household food expenditure and food expenditure outside the home (controlled) in the total sample (fixed effects panel models)

When the model was re-run on the total sample whilst controlling for individual fixed effects (income, lockdown, sex, age, education level, relationship status, number of children, occupation, UK region and year of data collection), the negative association between mental health and food expenditure became reversed such that higher (more favourable) GHQ scores were associated with higher household food expenditure (β = 0.95) (Table 3) and higher food expenditure outside the home (β = 0.49) (Table 4).

### 3.3 Household income

Higher (more favourable) GHQ scores were associated with higher household income in the models explaining household food expenditure (β = 0.71) and expenditure outside the home (β = 0.29). An interaction between income and food expenditure was calculated to determine if there was any associated change in mental health. The model indicated an interaction between income and household food expenditure (β = 0.03) and between income and food expenditure outside the home (β = 0.02). Higher household food expenditure (β = -0.11) and higher food expenditure outside the home (β = -0.05) were associated with lower GHQ scores and by increase in household income.

### 3.4 Pre/post lockdown

The controlled models explaining household food expenditure (Table 3) and food expenditure outside the home (Table 4) implied no differences in mental health pre and post lockdown. An interaction was calculated between pre-lockdown and post-lockdown to determine if there was any change in the association between mental health and food expenditure associated with the Covid-19 pandemic lockdown. A significant interaction was observed such that there was a significant positive relationship between mental health and household food expenditure pre-lockdown which changed to a negative relationship post-lockdown (β = -0.18) (Table 3). The association between more favourable mental health and higher household food expenditure altered post-lockdown, with higher household food expenditure becoming associated with less favourable mental health (Tables 1 and 2). There was no significant interaction between food expenditure outside the home and pre/post lockdown.

### 3.5 GHQ and food expenditure in white and non-white identities (fixed effects panel model)

The sample was sub-divided into those who reported a white identity and those who reported other identities (non-white). All who self-identified themselves as “White” in UKHLS survey were considered white and remainder who did not identify as “White” were considered non-White. When individual fixed effects were controlled in the model of household food expenditure, the positive association observed between GHQ scores (mental health) observed in the total sample was found to persist when the model was re-run exclusively on those who identified as white (β = 0.96). The relationship, however, disappeared when those who identified as non-white were considered (Table 3). When food expenditure outside the home was modelled (Table 4), the relationship to more favourable mental health again persisted in those who identified as white (β = 0.51) but was not evident among those with non-white identity.

### 3.6 Identity—Income

When household food expenditure (Table 3) was considered, more favourable mental health was associated with higher income among those who identified as white (β = 0.70) and those who identified as non-white (β = 0.69). When food expenditure outside the home was modelled (Table 4), income was associated with more favourable mental health in those who identified as white (β = 0.28), but not—those who identified as non-white. When the interaction between income and household food expenditure was applied, those who identified as white showed a negative relationship between income*food expenditure and mental health (β = -0.11). The food expenditure outside the home and mental health interaction model also founds a negative relationship between income*food expenditure outside home and mental health (β = -0.05) in those who identified as white. There was no interaction effect between food expenditure variables* and income upon GHQ scores among those who identified as non-white.

### 3.7 Identity—Pre/post lockdown

There was no relationship between mental health (GHQ scores) pre or post lockdown in either those who identified as white or non-white in either the model of household food expenditure or food expenditure outside the home. An interaction between lockdown dummy and food expenditure variables was introduced in the models to determine if there was any change in mental health and expenditure associated with the pandemic pre-lockdown and post-lockdown. No significant interaction effect was found between mental health pre/post lockdown in either those with a white or non-white identity when household expenditure or expenditure outside the home was modelled.

## 4. Discussion

Food prices have been increasing in the UK and causing concern among the general public [20] and has been reflected in increased food expenditure [1]. The aim of this analysis has been to inform and target interventions and policies around mental health and food expenditure. The objective has been to understand how changes in food expenditure relate to mental health taking income and lockdown into account and comparing in households of different ethnic identities. We hypothesised that there would be a relationship between mental health and food expenditure and that this association would be income dependent, change following the Covid-19 pandemic lockdown and vary in white and non-white identities.

As hypothesised, mental health was related to food expenditure. Initial analysis indicated that more favourable mental health was associated with decreasing food expenditure within and outside the home. The direction of the relationship between mental health and food expenditure, however, altered when socio-demographic factors were controlled, such that more favourable mental health became associated with greater household food expenditure and greater food expenditure outside the home. Conversely, households that spent less on food and sourced food outside the home less often, reported poorer mental health. This was as predicted given previous research indicating that between 2021 and 2022, UK household food expenditure increased while expendure outside the home decreased [16] and consistent with previous research indicating less favourable mental health among those experiencing difficulty in meeting the cost of household expenditure [13]. Lower food expenditure may be a response to increased demands on household budgets as a result of increased energy and other costs [17]. This result is also consistent with previous research indicating lower food expenditure in food insecure households [36] and inability to meet UK dietary recommendations to consume more than five portions of fruit and vegetables per day [28]. Further research is required to better understand the mechanisms underlying this association between lower food expenditure and less favourable mental health.

This observed relationship between mental health and food expenditure, as hypothesised, appeared to be income dependent. Less favourable mental health in lower income households was associated with greater food expenditure both within and beyond the household. This was as expected, given existing evidence for poorer mental health in people on lower incomes [10, 11, 13], and among those residing in households with less disposable income [10]. As food is a non-discretionary expense, those on lower incomes, spend a greater proportion of their income on food and are more financially vulnerable to increases in food prices [16]. Together, these findings affirm the notion that people in less affluent circumstances experience detriment to mental health [10, 11] and take this notion further to imply that this detriment can be exacerbated by greater food expenditure.

We also sought to determine any change in mental health and food expenditure with pandemic lockdowns. Given previous research, it was hypothesised that lockdowns would have impacted negatively upon mental health [37] and that this would be associated with increases in cost of food [16] and food expenditure [38, 39]. As expected, therefore, poorer mental health was associated with greater household food expenditure post-lockdown. No association was observed between mental health and food expenditure outside the home post-lockdown, possibly because people were eating out less often following the pandemic [2].

Also as predicted, the association between food expenditure and mental health differed between white and non-white identities. When those who identified as white and non-white were modelled separately, less favourable mental health was again associated with greater food expenditure both within and beyond the household, but only in those who identified as white. Mental health was unrelated to food expenditure both within and outside the household in those who identified as non-white. This agrees with previous research indicating that people in the UK of a white identity have poorer mental health and experience greater food insecurity [22]. This analysis suggests that households of white identity experience greater detriment to mental health when food expenditure is greater. Whilst food policy specifically targeted towards assisting households on lower household budgets may be needed, further research will be required to understand barriers to food security experienced by those who identify as white.

### 4.1 Strengths and limitations

Despite the GHQ having proven utility for assessing mental health in the general population [6–8] these findings should be interpreted with caution given mental health was measured on the unidimensional, short (12 item) version of the GHQ which may be less sensitive than longer tools in identifying clinically relevant mental health deficit [40]. The self-reported nature of the GHQ, with questions that can vary according to respondents’ perceptions of their mental health, means that the findings are not comparable to clinical evaluation from a physician. This implies that mental health in this sample may be even less favourable than indicated, rendering null findings related to gender unreliable. Household food expenditure measures are also limited in that they do not consider individual differences in spending. Another potential limitation is that, although we controlled for number of children, we did not equivalise household food expenditure to household size.

Other potential limitations relate to the sampling. That this analysis has only considered the responses of the head of household, who may or may not have been the main shopper, may have affected the strength of conclusions. Although ethnicity is a multifaceted, socially constructed entity [30], constraints imposed by official classifications employed in the understanding society survey [31], limited this analysis to a broad categorisation of identity as white/non-white. Another potential limitation is that the food expenditure measures may not be sensitive to cultural differences in the types of food purchased. That the food expenditure measures were not culturally sensitive, however, could render them more objective and amenable to generalisation across cultures.

Because data were collected by cross-sectional survey, albeit at successive time-points, we cannot conclude on the direction of cause and effect between mental health and food expenditure. The food expenditure variable may have been affected by inflation which we have not measured. Another limitation inherent in the food expenditure variable is that it comprised only two questions that were created by the authors of the survey and not validated specifically to assess food expenditure. The self-reported nature of the responses may have further confounded results. That we have not controlled for attrition or missingness may also have imposed limits on the ability to draw conclusions. That missingness was low at 1%, and given the large sample size, however, this is unlikely to have affected the results. Among strengths of this secondary analysis is that it draws on data derived from a large sample representative of the UK population rendering findings generalisable.

### 4.2 Conclusion

This analysis has sought to better understand how food expenditure relates to mental health. Mental health was found to be poorer where food expenditure was greater, particularly among those on lower incomes and in households identifying as white. Our findings suggest that food expenditure, income and ethnic identity should be considered when designing policies and programs to promote mental health. Policies and initiatives directed toward helping households, particularly those on low incomes, to reduce their food expenditure, could bring about a concurrent improvement in mental health. Given inability to determine the direction of cause and effect from these analyses, further research will be required to unpick and better understand the relationships we have identified between food expenditure and mental health. To maintain mental health, more stringent intervention may be needed to keep food prices down and reduce non-food spending targeted specifically toward lower income households. Fiscal policies are needed to widen access to free school meals, provide food vouchers and provide support for community gardens and food banks, all of which would alleviate food spending in lower income households. Food budgets are also likely to come under pressure from increased spending on non-food-related products and services so that the poorest households may be forced to buy less food, cheaper products and eat out less often to save money [28]. Policies are also needed to control and tackle the wider economic, societal pressures that indirectly affect food prices, and which squeeze food budgets. Putting caps on fuel/energy prices, providing help with childcare, housing and transport costs should help with food expenditure and thereby boost mental health. Meanwhile, these findings highlight the need for cross-sector collaboration on mental health and economic development policies directed toward achieving UN General Assembly (2012) Sustainable Development Goals [41] directed toward irradicating poverty and hunger, reducing inequality and improving mental health by 2030.
